# *Costus afer* Possesses Carbohydrate Hydrolyzing Enzymes Inhibitory Activity and Antioxidant Capacity *In Vitro*

**DOI:** 10.1155/2015/987984

**Published:** 2015-07-13

**Authors:** Armelle D. Tchamgoue, Lauve R. Y. Tchokouaha, Protus A. Tarkang, Jules-Roger Kuiate, Gabriel A. Agbor

**Affiliations:** ^1^Centre for Research on Medicinal Plants and Traditional Medicine, Institute of Medical Research and Medicinal Plants Studies, BP 6163, Yaoundé, Cameroon; ^2^Laboratory of Microbiology and Antimicrobial Substances, Department of Biochemistry, University of Dschang, P.O. Box 67, Dschang, Cameroon

## Abstract

Diabetes mellitus is a metabolic disorder of glucose metabolism which correlates with postprandial hyperglycemia and oxidative stress. Control of blood glucose level is imperative in the management of diabetes. The present study tested the hypothesis that *Costus afer*, an antihyperglycemic medicinal plant, possesses inhibitory activity against carbohydrate hydrolyzing enzymes. Hexane, ethyl acetate, methanol, and water extracts were prepared from the leaf, stem, and rhizome of *C. afer* and subjected to phytochemical screening, assayed for *α*-amylase and *α*-glucosidase inhibitory activities and antioxidant capacity (determined by total phenolic and total flavonoids contents, ferric reducing antioxidant power (FRAP), and DPPH radical scavenging activity). All extracts inhibited *α*-amylase and *α*-glucosidase activities. Ethyl acetate rhizome and methanol leaf extracts exhibited the best inhibitory activity against *α*-amylase and *α*-glucosidase (IC_50_: 0.10 and 5.99 mg/mL), respectively. Kinetic analysis revealed two modes of enzyme inhibition (competitive and mixed). All extracts showed antioxidant capacity, with hexane extracts exhibiting the best activity. DPPH assay revealed that methanol leaf, rhizome, and ethyl acetate stem extracts (IC_50_ < 5 mg/mL) were the best antioxidants. The presence of bioactive compounds such as flavonoids, alkaloids, phenols, and tannins may account for the antioxidant capacity and carbohydrate hydrolyzing enzyme inhibitory activity of *C. afer*.

## 1. Introduction

Diabetes mellitus (DM) remains the world's most common metabolic disorder resulting from defects in insulin secretion and/or action [1]. The global prevalence of diabetes is on the rise with at least 250 million individuals suffering from diabetes and a possible double by 2030. Earlier researchers have reported increased oxidative stress in sustained hyperglycemia characterized by increased free radical generation from increased glycation of proteins, autooxidation of glucose, and alterations in polyol pathway activity [2]. Free radicals, secreted by macrophages, T-cells, and natural killer cells as body defense, may still cause damage to *β*-cells [3]. Thus, plants or compounds with both hypoglycemic and antioxidant properties could be useful antidiabetic agents. The best therapeutic approach for diabetic complications will be targeting both glucose metabolism and the mechanisms of diabetes-induced oxidative stress.

Alpha-amylase and *α*-glucosidase inhibitors are drug-design targets in the development of compounds for the treatment of diabetes, obesity, and hyperlipemia [4]. Alpha-amylase secreted in saliva and pancreatic juice catalyzes the hydrolysis of starch to a mixture of smaller oligosaccharides consisting of maltose, maltotriose, and oligoglucans [5]. Alpha-glucosidase located in the mucosal brush border of the small intestine then degrades the oligosaccharides to glucose which is absorbed into the bloodstream [5]. Medicinal plants may constitute a good source of *α*-amylase and *α*-glucosidase inhibitors.* C. afer* is a useful medicinal plant that is highly valued for its antidiabetic, anti-inflammatory, antimicrobial, and antiarthritic properties and stomach complaints [6].

*C. afer*, of the Zingiberaceae family, commonly called bush sugar cane or monkey sugar cane [7], is a monocot and a relatively tall, herbaceous, unbranched tropical plant with creeping rhizome. It is commonly found in moist and shady forest of West and Tropical Africa [8].* C. afer* is a perennial, rhizomatous herb that can attain a height of up to 4 m. Leaves are simple and arranged spirally. Sheath is tubular, closed, and green with purple blotches; ligule is 4–8 mm long, leathery, and glabrous; petioles are 4–12 mm long; blade is elliptical to obovate, 15–35 cm × 3.5–9.5 cm, base is rounded to subcordate, apex is acuminate, and margin is sparsely hairy, usually glabrous above, sometimes shortly hairy beneath. Flowers are bisexual and zygomorphic [7].

The present study was designed to evaluate the enzyme inhibitory effects of extracts from different parts of* C. afer* using different solvents, against *α*-amylase and *α*-glucosidase, as well as their antioxidant activities, in view of the development of an appropriate phytomedicine for DM treatment.

## 2. Materials and Methods

### 2.1. Preparation of Plant Material

**Figure 1:**
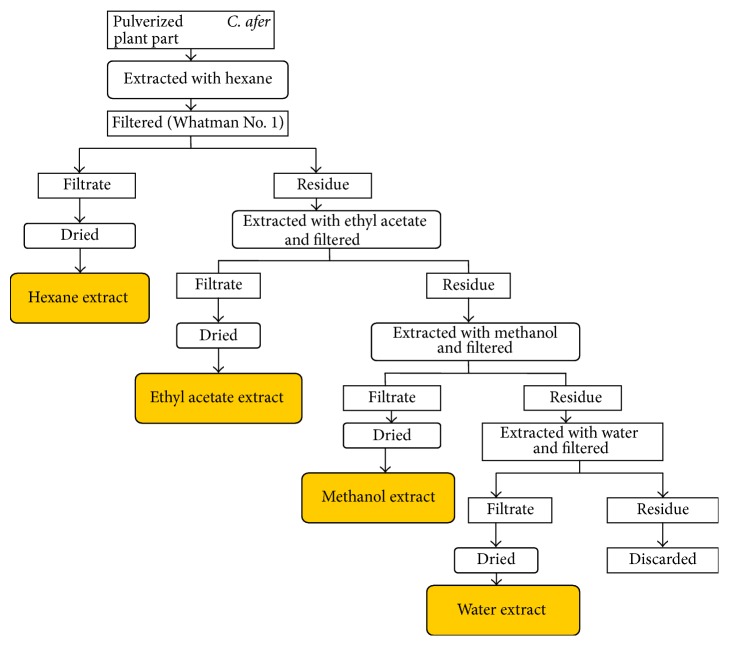
Schematic representation of the extraction of each of the parts of* Costus afer*.

Samples of leaf, stem, and rhizome of* Costus afer* (A. Rich.) were collected fresh from their natural habitat in Yaoundé, Cameroon, with the assistance of an ethnobotanist, Dr. Tsabang Nole. Samples were rinsed with tap water, chopped into small pieces, air-dried at room temperature, and then pulverized into fine powder. The pulverized samples were each sequentially extracted twice (Figure 1) with solvents of increasing polarity (hexane, ethyl acetate, methanol, and water) giving a total of 12 extracts (4 per plant part). The extracts were concentrated to reduce volumes using a rotavapor and finally powdered by evaporating the remaining solvents in a hot air oven at 40°C. All extracts were stored at −20°C until use.

### 2.2. Preliminary Phytochemistry Screening

A qualitative phytochemical screening was carried out to determine the presence of bioactive group of components such as alkaloids, flavonoid, saponin, anthraquinone, triterpenes, anthocyanin, tannins, steroids, glycosides, and phenols [9, 10].

#### 2.2.1. Test for Alkaloids

In a test tube containing 1 mL of extract, a few drops of Dragendorff's reagent were added and colour development was noticed. Appearance of orange colour indicates the presence of alkaloids.

#### 2.2.2. Test for Anthocyanins

Five drops of concentrated hydrochloric acid were added to the aqueous extract in a test tube and the change in color was observed; a red color indicated the presence of anthocyanins.

#### 2.2.3. Test for Flavonoids

Powdered plant material (1 g) was completely dissolved with acetone. The acetone extract was evaporated in a warm water bath and filtered while still hot, the filtrate cooled, and 5 mL of 20% NaOH added. A yellow solution indicated the presence of flavonoids.

#### 2.2.4. Test for Phenols

The test extract (100 mg) was dissolved in 3 mL of 70% ethanol. Three drops of 10% ferric (III) chloride were then added and the color change was observed. Appearance of a blue-violet colour indicated the presence of phenols.

#### 2.2.5. Test for Saponins

The plant extract (5 g) was shaken with water in a test tube. Frothing which persisted on warming was taken as preliminary evidence for the presence of saponins. A few drops of olive oil were added to 0.5 g of the extract and vigorously shaken. Formation of soluble emulsion in the extract indicated the presence of saponins.

#### 2.2.6. Test for Tannins

Water extract of the sample was treated with 15% ferric chloride test solution. The resultant color was observed. A blue color indicated the presence of hydrolysable tannins. A second confirmatory test was carried out: 0.5 g of the extract was added to 10 mL of freshly prepared potassium hydroxide in a beaker and shaken to dissolve. A dirty precipitate indicated the presence of tannins.

#### 2.2.7. Test for Triterpenes and Sterols (Liebermann Burchard Test)

The extract (100 mg) was dissolved in 3 mL of methanol and then 0.2 mL of each of chloroform, glacial acetic acid, and concentrated sulphuric acid was added. The solution was then observed for colour change; the appearance of a greenish blue or purple pink colour indicated the presence of sterols or triterpenes, respectively.

#### 2.2.8. Test of Glycosides

To a portion of the plant extract, 2 mL of glacial acetic acid and one drop of ferric chloride solution were added. Then 1 mL of concentrated sulphuric acid was added. A violet brownish ring below the interface followed by the formation of a greenish ring in the acetic acid layer indicated the presence of glycosides.

#### 2.2.9. Test of Anthraquinones

Few drops of hydrochloric acid (10%) were added to 2 g of extract in 10 mL of ether-chloroform mixture. After filtration, 1 mL of NaOH (10%) was added to 1 mL of filtrate; the appearance of red color indicated the presence of anthraquinones.

### 2.3. Determination of Total Phenolic Content (TPC)

The TPC of each extract was determined using Folin-Ciocalteu reagent with catechin used as the standard [11]. Into each test tube containing 980 *μ*L of Folin-Ciocalteu's reagent (diluted 5 times), 20 *μ*L of each extract (10.0 mg/mL) was added. The tubes were left at room temperature for 15 minutes, and the absorption was measured at 760 nm. Results obtained were expressed as catechin equivalents (CAE)/gm of plant material.

### 2.4. Total Flavonoid Content (TFC)

The method earlier described by Chang et al. [12] was applied in the estimation of TFC. Each plant extract (10.0 mg/mL) in methanol was separately mixed with 0.2 mL of 5% NaNO_2_. After 5 min, 0.2 mL of 10% AlCl_3_ was added and then 10 min later 2 mL of 1 M NaOH was added. The absorbance of the reaction mixture was measured at 510 nm 10 min later. The TFC was expressed as milligram/gram of rutin equivalent.

### 2.5. Ferric Reducing Antioxidant Power (FRAP)

The ferric reducing antioxidant power (FRAP) of extracts was determined as earlier described by Benzie and Strain [13]. The FRAP reagent consisted of ten parts of acetate buffer (300 mM, pH 3, 6), one part of 2,4,6-tripyridyl-s-triazine (TPTZ) (10 mM in 400 mM of HCl, Sigma), and one part of ferric chloride (10 mM). Briefly, each extract solution (75 *μ*L of 10.0 mg/mL) was added to 2 mL of FRAP reagent. The standard curve was prepared using catechin standard (50 *μ*M–600 *μ*M). The FRAP was expressed as milligram/gram of catechin equivalent. Catechin was used as the standard and absorbance read at 593 nm.

### 2.6. DPPH (2,2-Diphenyl-1-picrylhydrazyl) Free Radical Scavenging Assay

DPPH free radical scavenging assay was measured using DPPH free radical test, employing method of Blois [14]. The initial absorbance of DPPH in methanol was measured using spectrophotometer at 517 nm until the absorbance reading stabilized. A total of 100 *μ*L of each extract (10.0 mg/mL) was added to 900 *μ*L of 0.1 mM methanol DPPH solution. The mixture was incubated at room temperature in a dark cupboard for 30 min and the change in absorbance was measured at 517 nm. The percentage inhibition of the radical scavenging activity was calculated using the formula

(1) Percentage  inhibition  %=A517  of  control−A517  of  sampleA517  of  control×100.

The IC_50_ values were determined from plots of percent inhibition versus concentration of extracts.

### 2.7. Alpha-Amylase Inhibition Assay

The method earlier described by Conforti et al. [15] was used in this assay. A 0.5%, w/v, starch solution was prepared in 20 mM sodium phosphate buffer saline (pH 6.9) solution with heating for 15 min at 65°C. The color reagent was prepared by dissolving sodium potassium tartrate (12 g) in 8 mL of 2 M sodium hydroxide and 96 mM of 3,5-dinitrosalicylic acid solution. The assay protocol was as follows: 50 *μ*L of each extract at different concentrations (0.0625–2 mg/mL) and 50 *μ*L of *α*-amylase from* Aspergillus oryzae* (5 U/mL). After incubation of the mixture at 37°C for 15 min, 50 *μ*L of freshly prepared starch solution was added and further incubated for 20 min. 2 mL of stop/color reagent was added and the mixture was boiled for 15 min in a water bath. Acarbose was used as positive control. The absorbance was measured at 540 nm and the *α*-amylase inhibitory activity was calculated using the following equation:

(2) %  Inhibition=Absorbance  of  control−Absorbance  of  extractAbsorbance  of  control×100.

### 2.8. Alpha-Glucosidase Inhibitory Activity

The effect of the plant extracts on alpha-glucosidase activity was determined according to the chromogenic method described by Kim et al. [16] with slight modifications. The substrate solution p-nitrophenyl glucopyranoside (pNPG) was prepared in distilled water. Then phosphate buffer (20 mM, pH 6.9), 3 mM of glutathione reduced solution, and sodium carbonate (100 mM) were also prepared while 0.15 units of alpha-glucosidase (from* Bacillus stearothermophilus*) were preincubated with each extract of* C. afer* at different concentrations (0.0156 to 10 mg/mL) for 5 minutes. 10 mM of substrate (pNPG) was then added to start the reaction. The reaction mixture was incubated at 37°C for 20 minutes and stopped by adding 2 mL of 100 mM Na_2_CO_3_. The *α*-glucosidase activity was determined by measuring the yellow colored p-nitrophenol released from pNPG at 400 nm. Acarbose was used as the standard while phosphate buffer was used as control. The *α*-glucosidase inhibitory activity was calculated using the same equation (2).

The concentration of the extract that inhibited 50% of enzyme activity (IC_50_) was determined from plots of percent inhibition versus concentration.

### 2.9. Kinetics of Inhibition against *α*-Amylase and *α*-Glucosidase

Extracts with suitable IC_50_ values were selected for the inhibition kinetics study. The modes by which the selected extracts inhibited *α*-amylase and *α*-glucosidase activities were determined according to the method described by Kim et al. [16]. Briefly, fixed amounts of both *α*-amylase and *α*-glucosidase were incubated with increasing concentrations (0.08–5 mg/mL) of their substrates (starch and pNPG, resp.) at 37°C for 20 min, in the absence or presence of different extracts. Reactions were terminated and absorbance was read as previously mentioned. Amounts of products liberated (reducing sugars as maltose and p-nitrophenol, resp.) were determined from corresponding standard curves and converted to reaction rates according to the following formula: reaction rate (*V*) (mg/mL/s) = amount of product liberated (mg/mL)/1200 (s).

The mode of inhibition was determined by Lineweaver-Burk double reciprocal plot (1/*V* versus 1/[*S*]) using Michaelis-Menten kinetics, where* V* is the reaction velocity (reaction rate) and [*S*] is substrate concentration [17]. Kinetic parameters such as the Michaelis-Menten constant affinity (*K*_*m*_) and maximum velocity (*V*_max_) were derived from the plots.

### 2.10. Statistical Analysis

All results were expressed as mean ± SEM for triplicate determinations. Data were subjected to one-way analysis of variance (ANOVA) followed by Tukey's multiple comparison tests. Differences of *p* < 0.05 were considered statistically significant.

## 3. Results

### 3.1. Preliminary Phytochemical Screening

Phytochemical screening revealed that, with exception of the methanol stem and leaves extracts, anthocyanin, anthraquinones, and tannins were absent in the other extracts. Alkaloids, saponins, flavonoids, glycosides, and triterpenes were present in all plant parts solvent extracts (Table 1).

### 3.2. Antioxidant Properties

Four parameters were used for the evaluation of the antioxidant capacities of the different extracts: TPC, TFC, FRAP, and DPPH (Table 2). Irrespective of the plant part, water and methanol poorly extracted phenols and flavonoids because their total contents were significantly lower compared to the other extracting solvents. Hexane and ethyl acetate appear to be the suitable extracting solvents, showing higher TPC, TFC, and FRAP. The DPPH scavenging activity was negatively correlated to TPC (*r* = −0.466, *p* = 0.04), TFC (*r* = −0.638, *p* = 0.0001), and FRAP (*r* = −0.419, *p* = 0.001). FRAP was positively correlated to TPC (*r* = 0.725, *p* = 0.0001) and TFC (0.479, *p* = 0.001). Generally, these three parameters were comparable for the different plant parts irrespective of the extracting solvent. Considering the overall additional effect of extracts (hexane + ethyl acetate + methanol + water), the leaf extract had the best antioxidant capacity followed by the stem and then the rhizome extracts.

### 3.3. *In Vitro* Antidiabetic Activities

**Figure 2:**
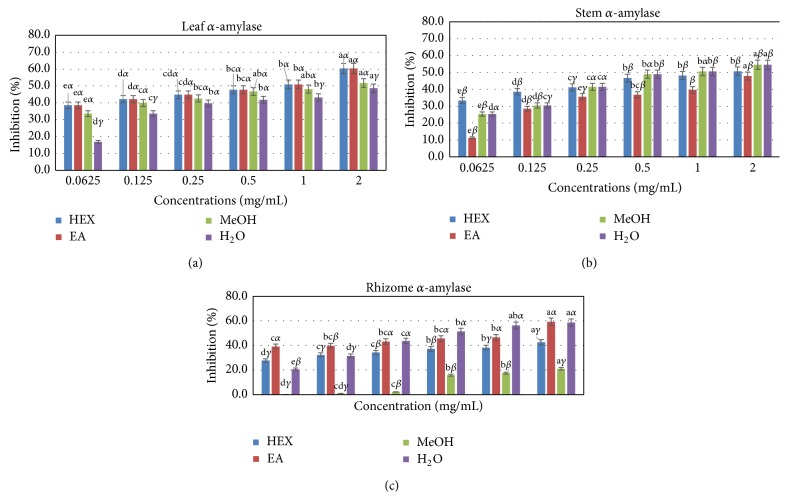
Inhibitory effect of solvent extracts from different parts of* C. afer* on *α*-amylase activities. Letters a, b, c, d, and e compare different solvents extracts at the same concentration and bars designated with different letters are significantly different from each other at *p* < 0.05 (Tukey HSD test). *α*, *β*, and *δ* compare the effect of concentration of the same solvent extract on *α*-amylase inhibitory activity and bars designated with different letters are significantly different from each other at *p* < 0.05 (Tukey HSD test).

**Figure 3:**
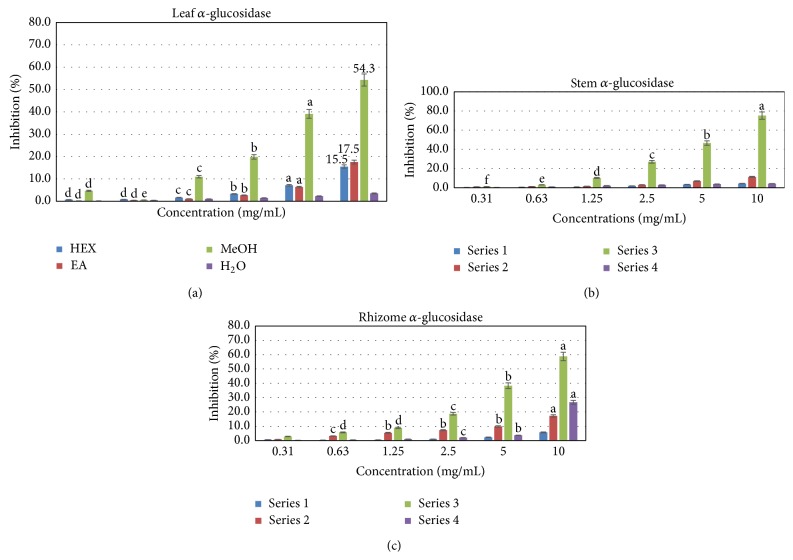
Inhibitory effect of methanol extracts from different parts of* C. afer* on *α*-glucosidase activities. Letters a, b, c, d, and e compare different solvents extracts at the same concentration and bars designated with different letters are significantly different from each other at *p* < 0.05 (Tukey HSD test).

The concentrations of the extracts that exhibited inhibitory effects on the enzymatic activity of *α*-glucosidase were higher compared to those with strong activity on the *α*-amylase (Figures 2 and 3). Only methanolic extracts showed significant inhibitory effect on the enzymatic activity of *α*-glucosidase. Its IC_50_ values, 5.9, 6.3, and 8.0 mg/mL, respectively, for leaf, stem, and rhizome extracts, were significantly lower compared to extracts obtained with other extracting solvents (Table 3). This effect was dose dependent irrespective of the plant part. In general, leaf extracts, stem, and rhizomes showed comparable effects (*p* > 0.05) on the enzymatic activity of *α*-glucosidase. It should be noted that the aqueous extract of rhizomes was more effective than the aqueous extracts of the two other parts of the plant indicating that inhibitors of this enzyme may be more concentrated in this plant part.

Regarding the *α*-amylase, virtually all samples, regardless of plant parts, showed quite pronounced inhibitory effects at the different concentrations tested. In general, the higher the concentration, the greater the inhibitory effect on *α*-amylase. For leaf and stem, hexane and methanolic extracts exhibited comparable inhibitory effects (*p* > 0.05), but significantly higher effects (*p* < 0.05) compared to rhizomes on *α*-amylase activity. With ethyl acetate extracts, the leaf and the rhizome showed comparable effect and more activity than the stem, while for the water extracts, leaf had intermediate activities comparable to those of rhizome and stem. The IC_50_ values of the tested extracts were lower for methanol rhizome extract (0.1 mg/mL), hexane (0.9 mg/mL), MeOH (0.6 mg/mL), and water (0.4 mg/mL) extracts from stem while leaf hexane and leaf acetate (0.7 mg/mL) were comparable. These values were lower than IC_50_ of the reference drug, acarbose (3.9 mg/mL). Thus, these extracts could inhibit these enzymes at much lower concentrations than even acarbose and would therefore be good candidates to test for high-affinity inhibitors. For this, inhibitor kinetic studies were performed on these selected extracts to determine the type of inhibition.

**Figure 4:**
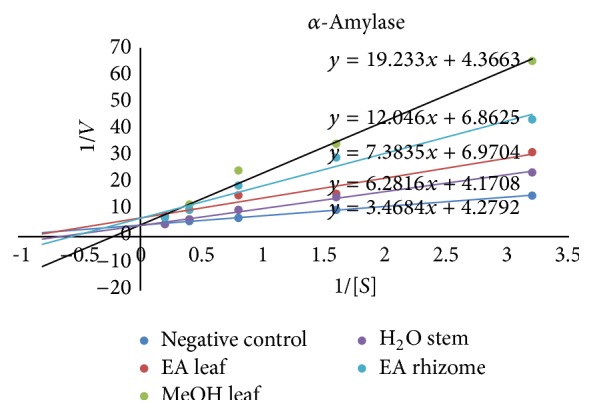
Lineweaver-Burk plots of activity of *α*-amylase from* Aspergillus oryzae* in the absence (control) or presence of the methanol and ethyl acetate leaf, water stem, and ethyl acetate rhizome extracts.

**Figure 5:**
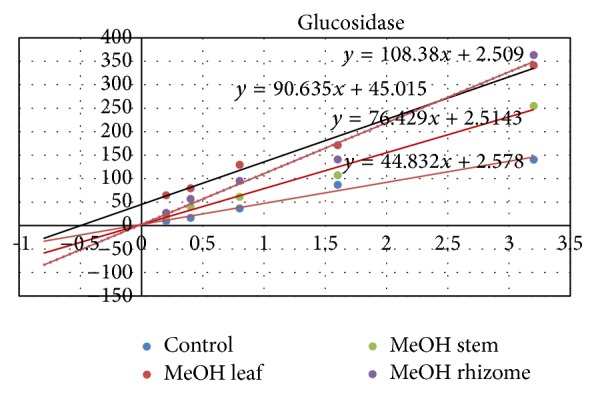
Lineweaver-Burk plots of activity of *α*-glucosidase from* Bacillus stearothermophilus* in the absence (control) or presence of the methanol leaf, stem, and rhizome extracts.

Among all the extracts, only methanol leaf extract inhibited both *α*-amylase and *α*-glucosidase. Ethyl acetate leaf extract inhibited *α*-amylase through a mixed mechanism with *V*_*m*_ and *K*_*m*_ different from those of the control (Figure 4, Table 4). Methanol leaf, water stem, and rhizome extracts exerted a competitive inhibition vis-à-vis this enzyme showing the same *V*_*m*_ and different *K*_*m*_ compared to the control (Figure 4). On *α*-glucosidase, it was observed that the methanol leaf extract showed mixed inhibitory mode while the methanol stem and rhizome extracts had a competitive inhibitory mode (Figure 5, Table 4).

## 4. Discussion

According to Nwauche et al. [18], the aqueous extract of* Costus afer* might possess insulin-like effect on the peripheral tissue either by promoting glucose uptake and metabolism or inhibiting hepatic gluconeogenesis. These properties were observed in an* in vivo* diabetic rat model. The present study revealed another possible mechanism of* Costus afer* on diabetes, based on glycolytic enzyme inhibition and antioxidant properties. Indeed, the extracts from different parts of this plant possess variable* in vitro* inhibitory effect on *α*-glucosidase and *α*-amylase, associated with a relatively high antioxidant capacity. These properties are linked to the phytochemical content of the different plant extracts. Inhibition of *α*-glucosidase and *α*-amylase is considered to be an efficient strategy in the treatment of carbohydrate metabolic disorders including diabetes mellitus type II [19]. Alpha-amylase is an endoglucanase secreted by the salivary gland and the pancreatic gland which hydrolyzes large insoluble starch (polysaccharides) to absorbable molecules (oligosaccharides and disaccharides), whereas *α*-glucosidase located at the surface of the membrane of the brush border of intestinal cells catalyzes the end step of digestion of starch and disaccharides [20]. Some herbal plant extracts have been reported for their *α*-amylase and *α*-glucosidase inhibitory activities [21] but to date no such activity has been reported for* C. afer.* These activities are a result of their bioactive components, which could be exploited in the management of diabetes [22].

Controlled kinetics of carbohydrate digestion and monosaccharide absorption could be of great value in the management of conditions such as diabetes. Thus, amylase and glucosidase inhibitors are of particular importance [23]. Only methanolic extracts showed a concentration-dependent inhibitory effect on *α*-glucosidase with lower activity for rhizomes while all other extracts showed relatively high inhibitory activities on *α*-amylase. These inhibitory properties may be partially due to alkaloids and sesquiterpenes that were absent in the less active rhizome methanolic extract. Ethyl acetate and methanol leaf extracts showed mixed inhibitory mechanism, respectively, on *α*-amylase and *α*-glucosidase versus competitive inhibitory effect of rhizomes and stem extracts. The bioactive components responsible for these properties are surely different from one extract to another and their chemical composition could be different. The different plants extracts screened in this study possess a potential bioinhibitor of these enzymes including alkaloids, flavones, and tannins [24].

In competitive inhibition, inhibitor and substrate compete to bind on the same active site. High substrate concentration prevents inhibitor fixation making the inhibitor more efficient only at low substrate concentrations [25]. This type of inhibition is not suitable in treatment of diabetes. In mixed inhibition, inhibitor and substrate bind at different sites on the enzyme and the inhibitory efficiency is observed at low and high substrate concentrations. Mixed inhibitor has equal affinities for the free enzyme and the enzyme substrate complex and does not affect the binding of the substrate [26]. Thus, methanol leaf extract may be the best candidate for reducing the activity of *α*-glucosidase. Also Griffiths and Moseley [27] reported that polyphenolic compounds in plants inhibit the activities of digestive enzymes because of their ability to bind with proteins. Green tea polyphenols have also been reported to inhibit the activities of *α*-glucosidase and sucrose [28], while sweet potato polyphenols inhibit the activities of *α*-glucosidase [29], and berry polyphenols and flavonoids inhibit the activities of *α*-glucosidase and *α*-amylase [30]. Triterpenes [31] and alkaloids have also been reported to possess inhibitory activity against *α*-glucosidase and *α*-amylase activities.

Phytochemical studies revealed the presence of several bioactive compounds including alkaloids, flavonoids, tannins, saponins, glycosides, and phenols which could be responsible for the medicinal properties of* C. afer*. Some of these bioactive components possess antioxidant activities and antidiabetic activities. This indicates that* C. afer* in inhibiting enzymes in the gut may also fight against oxidative stress in the cells of the diabetic patient. These inhibitory activities correlated with a concentration effect on their antioxidant capacities. This is suggestive that the antioxidant capacities contributed to the modification of the carbohydrate metabolizing enzyme activities. A positive relationship between the antioxidant (total polyphenol and flavonoid content) capacity and inhibition of intestinal *α*-glucosidase and *α*-amylase has been previously reported [32–35], which might be the case in this study. Like earlier mentioned, polyphenols have the potential of inhibiting carbohydrate metabolizing enzymes (*α*-glucosidase and *α*-amylase) because of their ability to bind with proteins [36, 37]. We also observed that while the extracts were potent inhibitors of *α*-amylase activity (low IC_50_), even better than the reference drug acarbose, they could only inhibit *α*-glucosidase activity weakly (very high IC_50_). These findings are consistent with previous results in which polyphenols and flavonoids were reported to inhibit the activities of *α*-glucosidase and *α*-amylase with different affinities; while the latter were potent inhibitors of *α*-amylase, they only possessed weak inhibitory activities against *α*-glucosidase [32, 38].

## 5. Conclusion

Generally, in the present study, the different solvent extracts of all parts of* C. afer* constitute an array of bioactive constituents and possess antioxidant capacity which correlated to inhibitory activities against *α*-amylase and *α*-glucosidase. However, ethyl acetate and methanol leaf extracts, with mixed inhibitory activities on these enzymes, may be more suitable for the management of diabetes. Thus,* C. afer* may be a good source of natural antioxidants and potent inhibitor of *α*-amylase and *α*-glucosidase, associated with the insulin-like properties previously observed. These findings justify and support the use of this plant species in the treatment of diabetes.

## Figures and Tables

**Table 1:** Distribution of bioactive components in the different parts of *Costus afer*.

Part plant	Extract	Bioactive components									
		FLA	ALK	ST	PHE	ANTC	ANTH	SAP	Gly	TAN	TRI
Leaves	Hexane	+	+	+	+	−	−	−	+	−	+
	EA	+	−	+	−	−	−	−	+	−	+
	MeOH	+	+	+	+	−	−	+	+	+	+
	H_2_O	+	+	−	+	−	−	+	+	−	+

Stem	Hexane	+	+	+	−	−	−	−	−	−	+
	EA	+	+	+	−	−	−	−	+	−	+
	MeOH	+	+	+	+	−	−	+	+	−	+
	H_2_O	+	+	+	−	−	−	+	+	−	+

Rhizomes	Hexane	−	+	+	−	−	−	−	+	−	+
	EA	+	−	+	−	−	−	−	+	−	+
	MeOH	+	−	−	+	−	−	+	+	−	+
	H_2_O	+	−	−	−	−	−	+	+	−	+

+: presence; −: absence; FLA: flavonoids; ALK: alkaloids; ST: steroids; PHE: phenols; ANTC: anthocyanin; ANTH: anthraquinones; SAP: saponin; Gly: glycosides; TAN: tannins; TRI: triterpenes.

**Table 2:** Extraction solvent effect on the antioxidant capacity of different parts of *Costus afer*.

Plants parts	Solvent	TPC (mg/g cat. eqv.)	TFC (mg/g rutin eqv.)	FRAP (mg/g cat. eqv.)	DPPH (IC_50_)
Leaf	HEX	32.04 ± 1.44^a*α*^	18.18 ± 1.19^a*α*^	138.56 ± 0.8^b*α*^	7.69 ± 0.07^b*α*^
	Ethyl acetate	22.36 ± 2.48^b*β*^	15.42 ± 0.72^b*α*^	123.03 ± 0.26^c*δ*^	13.28 ± 0.43^b*β*^
	MeOH	10.5 ± 0.13^c*α*^	2.67 ± 0.06^c*α*^	251.84 ± 2.19^a*α*^	0.19 ± 0.03^c*α*^
	H_2_O	4.35 ± 0.48^d*α*^	1.43 ± 0.04^d*α*^	57.76 ± 0.14^d*α*^	59.07 ± 2.72^a*δ*^

Stem	HEX	25.12 ± 0.12^b*β*^	13.48 ± 0.02^a*β*^	114.88 ± 0.7^b*β*^	7.7 ± 0.08^c*α*^
	Ethyl acetate	29.83 ± 2.65^a*α*^	2.40 ± 0.08^b*β*^	156.89 ± 0.68^a*α*^	0.41 ± 0.06^d*α*^
	MeOH	9.34 ± 0.26^c*β*^	0.79 ± 0.02^d*β*^	103.04 ± 1.75^b*β*^	38.94 ± 0.49^a*δ*^
	H_2_O	2.56 ± 0.19^d*δ*^	1.11 ± 0.02^c*β*^	46.93 ± 0.76^c*β*^	17.23 ± 0.16^b*α*^

Rhizomes	HEX	31.67 ± 0.61^a*α*^	18.07 ± 0.95^a*α*^	105.3 ± 0.73^c*β*^	5.10 ± 0.03^c*β*^
	Ethyl acetate	20.83 ± 0.91^b*β*^	13.73 ± 0.04^b*β*^	139.43 ± 0.37^a*β*^	11.96 ± 0.0^b*β*^
	MeOH	3.72 ± 0.29^c*δ*^	2.21 ± 0.01^c*α*^	117.51 ± 0.52^b*β*^	4.92 ± 0.2^c*β*^
	H_2_O	3.37 ± 0.06^d*β*^	0.97 ± 0.01^d*β*^	55.04 ± 0.35^d*α*^	40.68 ± 2.2^a*β*^

^a,b,c,d^Solvents effect on antioxidant capacity of plant parts. Means with different letters (a, b, c, and d) within a column of the same plant part are significantly different from each other at *p* < 0.05. *αβδ* compares the antioxidant capacity of plant parts extracted with the same solvent. Means designated with different symbols are significantly different from one another at *p* < 0.05.

**Table 3:** Inhibitory concentration (IC_50_ (mg/mL)) of effective extracts of *C. afer* on *α*-amylase and *α*-glucosidase.

Plants parts/standard	Solvent extracts	*α*-Amylase	*α*-Glucosidase
Standard	Acarbose	3.92 ± 0.23	0.12 ± 0.06

Leaf	HEX	0.78 ± 0.10^a^	32.8 ± 1.22^b^
	EA	0.77 ± 0.08^a^	26.21 ± 0.43^b^
	MeOH	4.34 ± 1.08^b^	5.99 ± 0.07^a^
	H_2_O	4.91 ± 0.42^b^	176.88 ± 18.43^c^

Stem	HEX	0.99 ± 0.24^b^	124.88 ± 17.59^c^
	EA	1.74 ± 0.34^c^	49.23 ± 0.93^b^
	MeOH	0.65 ± 0.24^a,b^	6.31 ± 0.23^a^
	H_2_O	0.49 ± 0.08^a^	166.64 ± 9.41^d^

Rhizome	HEX	2.29 ± 0.42^c^	78.57 ± 6.3^d^
	EA	0.10 ± 0.01^a^	33.35 ± 2.09^c^
	MeOH	1.78 ± 0.04^b,c^	8.02 ± 0.02^a^
	H_2_O	1.42 ± 0.42^b^	14.62 ± 0.46^b^

^a,b,c,d^Solvents effect on inhibitory concentration of plant parts. Means with different letters (a, b, c, and d) within a column of the same plant part are significantly different from each other at *p* < 0.05.

**Table 4:** Kinetic parameters of effective extracts of *Costus afer* on alpha-amylase and alpha-glucosidase activities *in vitro*.

Plant parts	Extracts	Alpha-amylase		Alpha-glucosidase	
		*V*_max⁡_ (mg/mL/s)	*K*_*m*_ (mg/mL)	*V*_max⁡_ (mg/mL/s)	*K*_*m*_ (mg/mL)
	Control	0.23	0.81	0.39	17.39

Leaf	EA	0.14	1.06	nd	nd
	MeOH	0.23	4.43	0.02	2.01

Stem	MeOH	Nd	nd	0.39	30.4
	H_2_O	0.24	1.51	nd	nd

Rhizome	EA	0.23	3.76	nd	nd
	MeOH	Nd	nd	0.39	43.2

nd: not determined.

## References

[1] Moore H., Summerbell C., Hooper L. (2004). Dietary advice for treatment of type 2 diabetes mellitus in adults. *Cochrane database of systematic reviews (Online)*.

[2] Araki E., Nishikawa T. (2010). Oxidative stress: a cause and therapeutic target of diabetic complications. *Journal of Diabetes Investigation*.

[3] Kokil G. R., Rewatkar P. V., Verma A., Thareja S., Naik S. R. (2010). Pharmacology and chemistry of diabetes mellitus and antidiabetic drugs: a critical review. *Current Medicinal Chemistry*.

[4] Franco O. L., Rigden D. J., Melo F. R., Grossi-de-Sá M. F. (2002). Plant *α*-amylase inhibitors and their interaction with insect *α*-amylases. *European Journal of Biochemistry*.

[5] Gomathi D., Kalaiselvi M., Uma C. (2012). In vitro *α*-amylase and *α*-glucosidase inhibitory effects of ethanolic extract of *Evolvulus alsinoides* (L.). *International Research Journal of Pharmacy*.

[6] Soladoye M. O., Oyesika O. O. (2008). *A Textbook of Medicinal Plants From Nigeria*.

[7] Nyananyo B. L. (2006). Plants from the Niger Delta. *International Journal of Pure and Applied Sciences*.

[8] Iwu M. M. (2009). Traditional Igbo medicine. *Applied Sciences*.

[9] Trease G. E., Evans W. C. (1989). *Phamacognosy*.

[10] Sofowora A. (1993). Screening plants for bioactive agents. *Medicinal Plants and Traditional Medicine in Africa*.

[11] Vinson J. A., Yong A., Xuelci S., Ligid Z., Bose P. (2001). Phenol antioxidant and quantity and quality in foods. *Journal of Agriculture and Food Chemistry*.

[12] Chang L.-W., Yen W.-J., Huang S. C., Duh P.-D. (2002). Antioxidant activity of sesame coat. *Food Chemistry*.

[13] Benzie I. F. F., Strain J. J. (1996). The ferric reducing ability of plasma (FRAP) as a measure of ‘antioxidant power’: the FRAP assay. *Analytical Biochemistry*.

[14] Blois M. S. (1958). Antioxidant determinations by the use of a stable free radical. *Nature*.

[15] Conforti F., Statti G., Loizzo M. R., Sacchetti G., Poli F., Menichini F. (2005). *In vitro* antioxidant effect and inhibition of *α*-amylase of two varieties of *Amaranthus caudatus* seeds. *Biological and Pharmaceutical Bulletin*.

[16] Kim Y.-M., Jeong Y.-K., Wang M.-H., Lee W.-Y., Rhee H.-I. (2005). Inhibitory effect of pine extract on *α*-glucosidase activity and postprandial hyperglycemia. *Nutrition*.

[17] Lineweaver H., Burk D. (1934). The determination of enzyme dissociation constants. *Journal of the American Chemical Society*.

[18] Nwauche K. T. G., Monago C. C., Anacletus F. C. (2014). Antihyperglycemic activity of the aqueous extract of *Costus afer* stem alone and in combination with metformin. *European Journal of Biotechnology and Bioscience*.

[19] Floris A. L., Peter L. L., Reinier P. A., Elov H. L., Guy E. R., Chris W. (2005). Glucosidase inhibitors for patients with type 2 diabetes. *Diabetes Care*.

[20] Bougatef A., Hajji M., Balti R., Lassoued I., Triki-Ellouz Y., Nasri M. (2009). Antioxidant and free radical-scavenging activities of smooth hound (*Mustelus mustelus*) muscle protein hydrolysates obtained by gastrointestinal proteases. *Food Chemistry*.

[21] Picot C. M. N., Subratty A. H., Mahomoodally M. F. (2014). Inhibitory potential of five traditionally used native antidiabetic medicinal plants on *α*-amylase, *α*-glucosidase, glucose entrapment, and amylolysis kinetics in vitro. *Advances in Pharmacological Sciences*.

[22] Kunyanga C. N., Imungi J. K., Okoth M. W., Biesalski H. K., Vadivel V. (2012). Total phenolic content, antioxidant and antidiabetic properties of methanolic extract of raw and traditionally processed Kenyan indigenous food ingredients. *LWT—Food Science and Technology*.

[23] Kim S.-D., Hong J. N. (2004). Isolation and characterization of *α*-glucosidase inhibitor from the fungus *Ganoderma lucidum*. *Journal of Microbiology*.

[24] Alagesan K., Raghupathi P. K., Sankarnarayana S. (2012). Amylase inhibitors: potential source of anti-diabetic drug discovery from medicinal plants. *International Journal of Pharmacy and Life Sciences*.

[25] Rakesh S., Sharma R. (2012). Enzyme inhibition: mechanisms and scope. *Enzyme Inhibition and Bioapplications*.

[26] Copeland R. A., Harpel M. R., Tummino P. J. (2007). Targeting enzyme inhibitors in drug discovery. *Expert Opinion on Therapeutic Targets*.

[27] Griffiths D. W., Moseley G. (1980). The effect of diets containing field beans of high or low polyphenolic content on the activity of digestive enzymes in the intestines of rats. *Journal of the Science of Food and Agriculture*.

[28] Hara Y., Honda M. (1992). Inhibition of rat small intestinal sucrose and alpha-glucosidase activities by tea polyphenol. *Bioscience, Biotechnology, and Biochemistry*.

[29] Matsui T., Ueda T., Oki T., Sugita K., Terahara N., Matsumoto K. (2001). *α*-glucosidase inhibitory action of natural acylated anthocyanins. 1. Survey of natural pigments with potent inhibitory activity. *Journal of Agricultural and Food Chemistry*.

[30] McDougall G. J., Stewart D. (2005). The inhibitory effects of berry polyphenols on digestive enzymes. *BioFactors*.

[31] Meshram G. A., Khamkar S. S. (2014). Effect of oleanolic acid isolated from garlic leaves on carbohydrate metabolizing enzymes, in vitro. *International Journal of Pharmaceutical Sciences and Research*.

[32] McDougall G. J., Shpiro F., Dobson P., Smith P., Blake A., Stewart D. (2005). Different polyphenolic components of soft fruits inhibit *α*-amylase and *α*-glycosidase. *Journal of Agricultural and Food Chemistry*.

[33] Tadera K., Minami Y., Takamatsu K., Matsuoka T. (2006). Inhibition of *α*-glucosidase and *α*-amylase by flavonoids. *Journal of Nutritional Science and Vitaminology*.

[34] Mai T. T., Thu N. N., Tien P. G., van Chuyen N. (2007). Alpha-glucosidase inhibitory and antioxidant activities of Vietnamese edible plants and their relationships with polyphenol contents. *Journal of Nutritional Science and Vitaminology*.

[35] Ramkumar K. M., Thayumanavan B., Palvannan T., Rajaguru P. (2010). Inhibitory effect of *Gymnema montanum* leaves on *α*-glucosidase activity and *α*-amylase activity and their relationship withpolyphenolic content. *Medicinal Chemistry Research*.

[36] Hanamura T., Hagiwara T., Kawagishi H. (2005). Structural and functional characterization of polyphenols isolated from acerola (*Malpighia emarginata* DC.) fruit. *Bioscience, Biotechnology and Biochemistry*.

[37] Bothon F. T., Debiton E., Avlessi F., Forestier C., Teulade J.-C., Sohounhloue D. K. (2013). In vitro biological effects of two anti-diabetic medicinal plants used in Benin as folk medicine. *BMC Complementary and Alternative Medicine*.

[38] Padilla-Camberos E., Lazcano-Díaz E., Flores-Fernandez J. M., Owolabi M. S., Allen K., Villanueva-Rodríguez S. (2014). Evaluation of the inhibition of carbohydrate hydrolyzing enzymes, the antioxidant activity, and the polyphenolic content of citrus limetta peel extract. *The Scientific World Journal*.

